# Rapid α-Al_2_O_3_ Growth on an Iron Aluminide Coating at 600 °C in the Presence of O_2_, H_2_O, and KCl

**DOI:** 10.1021/acsami.4c11719

**Published:** 2024-10-17

**Authors:** Alina Agüero, Pauline Audigié, Sergio Rodríguez, Marcos Gutiérrez del Olmo, Jon Pascual, Vicent Ssenteza, Torbjörn Jonsson, Lars-Gunnar Johansson

**Affiliations:** † Área de Materiales Metálicos, Instituto Nacional de Técnica Aeroespacial, Carretera de Ajalvir Km 4, 28850 Torrejón de Ardoz, Spain; ‡ Department of Chemistry and Chemical Engineering, Chalmers University of Technology, Energy and Materials, Kemivägen 10, 412 96 Gothenburg, Sweden

**Keywords:** α-Al_2_O_3_, biomass-fired power plants, coatings, iron aluminide, ferritic steels, high-temperature corrosion

## Abstract

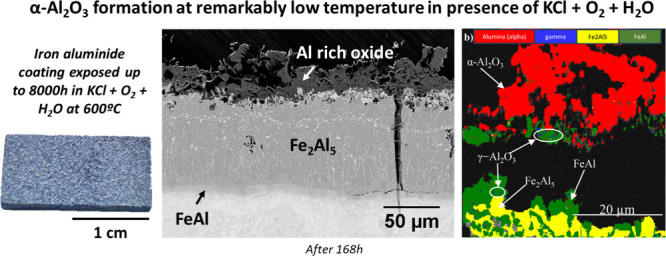

In this work, a slurry iron aluminide-coated ferritic steel SVM12 was subjected to a laboratory experiment mimicking superheater corrosion in a biomass-fired power boiler. The samples were exposed under model Cl-rich biomass conditions, in a KCl + O_2_ + H_2_O environment at 600 °C for 168, 2000, and 8000 h. The morphology of corrosion and the composition of the oxide scale and the coating were investigated by a combination of advanced analytical techniques such as FESEM/EDS, SEM/EBSD, and XRD. Even after short-term exposure, the coating developed a very fast-growing and up to 50 μm thick α-Al_2_O_3_ scale in contrast to the spontaneous formation of a protective, thin, dense, slow-growing, and very adhesive α-Al_2_O_3_ layer usually formed on metallic materials after high-temperature oxidation. In view of the literature on the formation of oxide scales on alloys and coatings, the formation of an α-Al_2_O_3_ scale at this relatively low temperature is very surprising in itself. The thick alumina scale was not protective as its formation resulted in fast degradation of the coating and rapid Fe_2_Al_5_ → FeAl phase transformation, which in turn generated porosity inside the coating. In all cases, the resulting thick Al_2_O_3_ scale was porous and consisted of both equiaxed α-Al_2_O_3_ grains and randomly oriented aggregated alumina whiskers. Potassium is concentrated in the outer part of the Al_2_O_3_ scale, while chlorine is concentrated close to the scale/aluminide interface. The unexpected formation of rapidly growing α-Al_2_O_3_ at relatively low temperature is attributed to the hydrolysis of aluminum chloride generated in the corrosion process.

## 1 Introduction

Al_2_O_3_ has several crystalline polymorphs, including the thermodynamically stable α-Al_2_O_3_ (corundum) and several metastable crystalline forms, i.e., γ–, θ–, η-, κ-, χ- and δ- Al_2_O_3_.^1,2^ α-Al_2_O_3_ is a refractory dielectric and is widely used as an oxide ceramic at high temperatures and for many other purposes, including as an abrasive, for prostheses, and, in single crystal form, as “sapphire glass”. The metastable aluminas form powders with very large surface area, and γ-Al_2_O_3_ is widely used in catalysis and as an adsorbent. Many alloys are designed to spontaneously form protective Al_2_O_3_ surface layers (Al_2_O_3_ scales) at high temperature in oxygen-containing gases, e.g., O_2_, H_2_O, and CO. Al_2_O_3_ forming materials and coatings are used in a wide variety of industrial high-temperature applications, including resistance heating, chemical and petrochemical plants, aeronautics, and gas turbines. The Al_2_O_3_ formers include FeCrAl, NiCrAl, and CoCrAl alloys, Al_2_O_3_ forming austenitic stainless steels, and transition metal aluminides.

Al_2_O_3_ forming alloys are usually used at relatively high temperatures, typically >800 °C. At lower temperatures the supply of Al to the growing Al_2_O_3_ scale, by diffusion in the substrate, may be insufficient for a continuous Al_2_O_3_ layer to form. Also, the most desirable protective oxide, α-Al_2_O_3_, forms at high temperatures. During early oxidation and <900 °C, metastable aluminas, i.e., γ- and θ-Al_2_O_3_, tend to form rather than α-Al_2_O_3_. This is not desirable because the metastable aluminas grow faster, and their transformation to α-Al_2_O_3_ generates stresses in the alumina scale. Because of the sluggish nucleation of α-Al_2_O_3_, Al_2_O_3_ forming alloys exposed below 900–1000 °C typically form surface oxide layers consisting of metastable (transient) aluminas (i.e., γ- and θ-Al_2_O_3_) which are less protective against corrosion than α-Al_2_O_3_.^3^ For instance, Brumm and Grabke^1^ reported that oxidation of β-NiAl in air resulted in γ- and θ-Al_2_O_3_ formation below 1050 °C while a minimum of 950 °C was needed to form α-Al_2_O_3_. In addition to growing faster on the alloy surface, oxide scales made up of transient Al_2_O_3_ polymorphs are also reported to be more permeable to gases, making the alloy susceptible to, e.g., nitridation.^4^

Lots of studies were devoted to the formation of α-Al_2_O_3_ on Al_2_O_3_ forming alloys and coatings for diverse applications at high temperature. Twenty years ago, Quaddakers et al. investigated the growth rates of alumina scales on Fe–Cr–Al alloys doped with reactive elements in the temperature range 1000–1300 °C in air^5^ and reported that α-Al_2_O_3_ was the main oxide formed. When they studied the oxidation behavior of Al_2_O_3_-forming ODS superalloys at 900 °C, the same group observed the influence of both the θ → α transformation and the presence of reactive element (yttrium) on the growth kinetics.^6^ Chevalier et al. characterized the alumina scales formed on FeCrAl alloys oxidized at even lower temperature and reported for the first time the stratification of γ-, θ-, and α-Al_2_O_3_ on an FeCrAl alloy after 100 h at 850 °C in air.^7^ Finally, Josefsson et al., who investigated the oxidation of FeCrAl alloys in air between 500 and 900 °C in dry and wet O_2_, report that the minimum temperature needed to form α-Al_2_O_3_ was 700 °C.^8^

Concerning Al_2_O_3_-forming coatings, aluminide diffusion and overlays coatings are particularly known to be used in thermal barrier coatings systems for their ability to sustain the formation of the protective α-Al_2_O_3_ thermally grown oxide (TGO) at temperatures higher than 1000 °C.^9−11^ Daroonparvar et al. studied the growth rate of some MCrAlY HVOF coatings in air at 1100 and 1150 °C and observed the formation of α-Al_2_O_3_ after 48 h. They also stated that a higher porosity and oxygen content in thermally sprayed APS and HVOF coatings can be associated with higher oxide growth rates.^11,12^ Coatings for power plants have also been investigated. As an example, Singh et al. studied the performance of HVOF Ni_3_Al coating on T22 steel in Na_2_SO_4_–60% V_2_O_5_ molten salt at 900 °C for 50 cycles of 1 h for a coal-fired boiler and observed the formation of Al_2_O_3_ along with NiO and Fe_2_O_3_ unprotective oxides after 50 cycles but without specifying the phase identity of Al_2_O_3_.^13^ All of the previously mentioned studies reflect the formation of α-Al_2_O_3_. However, none of them report the formation of thick α-Al_2_O_3_ scales. Indeed, they do not at all report on α-Al_2_O_3_ formation at temperatures as low as 600 °C. As an exception, α-Al_2_O_3_ has been reported to form on Fe–aluminide coatings exposed to pure steam at 650 °C. Initially, protective χ-Al_2_O_3_ formed which then transformed into α-Al_2_O_3_ after several hundred hours.^14^ Presently, Al_2_O_3_-forming FeCrAl alloys are being developed for applications at somewhat lower temperature.^4,15−18^ For instance, Sand et al. evidenced the formation of an inner dense oxide of α-Al_2_O_3_ on the surface of a Cr and Al-lean FeCrAl alloy doped with silicon at 800 °C in wet air while exposure at 600 °C led to the formation of Fe-rich nodules.^19^ Slurry-applied Fe aluminide coatings of the type dealt with in ref (14) have been explored for protection of steels in harsh high-temperature environments such as coal combustion gases,^20^ molten salts,^21−23^ steam,^24−26^ and high carbon activity (metal dusting) environments,^27,28^ among others, at temperatures between 580 and 700 °C. Also, aluminide coatings are being explored as alternatives to protect low-cost ferritic steels used in biomass-fired power boilers at around 600 °C. While the formation of α-Al_2_O_3_ scales would be desirable, metastable aluminas or even other oxides and spinels are expected to form in such applications, because of the low temperature.

Moreover, to achieve the lowest possible oxidation kinetics, the formation of an α-Al_2_O_3_ layer with a large grain size should be favored,^29,30^ as this reduces the diffusion at the grain boundaries. In terms of stress, it is also preferable to form an oxide scale with a uniform columnar morphology along the coating surface to ensure good adhesion between the coating and the substrate.

In any case, the interest in extending the application of Al_2_O_3_ forming materials to lower temperatures makes it worthwhile to investigate the morphology and phase composition of the oxide scales formed under such conditions.

The present paper reports on the novel observation of the formation of very thick α-Al_2_O_3_ scales on an iron aluminide coating in a laboratory experiment mimicking superheater corrosion in a biomass-fired power boiler at very low temperature (600 °C). The coated samples were exposed to an O_2_ + H_2_O environment in the presence of KCl at 600 °C for 168, 2000, and 8000 h. The morphology and composition of the scales are described. Possible causes behind the formation of α-Al_2_O_3_ at this surprisingly low temperature are discussed.

## 2 Experimental Procedure

### 2.1 Material and Coating Deposition

Substrate samples, with dimensions 20 mm × 10 mm × 2 mm, were machined from the wall thickness of tubes made from ferritic–martensitic steel Super VM12 (X13CrCoWMoVNbBN11-2-2; SEL.-No.: 1.4965) supplied by Vallourec S.A. The nominal composition of Super VM12 or SVM12 steel is detailed in Table 1.

**Table 1:** Nominal Composition of Super VM12 Steel (wt % and at. %)

		Fe	C	Mn	Si	Cr	Mo	V	Ni	B	N	Co	W	Nb
wt %	min	bal	0.10	0.30	0.20	10.50	0.10	0.15	0.10	0.008	0.002	1.50	1.50	0.02
	max		0.16	0.80	0.60	12.00	0.60	0.30	0.40	0.015	0.020	2.50	2.50	0.10
at. %	min	bal	0.56	0.30	0.40	11.26	0.06	0.16	0.09	0.04	0.01	1.42	0.45	0.01
	max		0.89	0.81	1.19	12.88	0.35	0.33	0.38	0.08	0.08	2.37	0.76	0.06

The coupons were ground with P180 SiC paper and degreased with acetone in an ultrasonic bath before applying the slurry coating. The slurry was prepared by mixing Al powder of 4–6 μm diameter (Poudres Hermillon, France) with water along with a proprietary Cr^6+^ free binder formed by inorganic compounds and was stirred several hours before use. Then, a 100–150 μm thick layer of slurry was sprayed with a Sagola 475 Xtech spray gun onto all of the faces of the ground Super VM12 coupons. Samples were then dried under laboratory air before being subjected to a diffusion heat treatment of 4 h at 760 °C under argon. Undiffused residues of the slurry, also named “bisque”, were removed by slight grinding.

### 2.2 Biomass Corrosion Testing

Coated samples were tested in the as-received state. To remove grease and dirt after grinding or coating, all samples were immersed in acetone in an ultrasonic bath for 5 min. The samples were then washed with ethanol. Prior to exposure, all samples were measured with a digital caliper and weighed with a precision balance (five decimals). After testing, the mass gain of the samples was determined.

The laboratory biomass corrosion test was designed based on results obtained in a preliminary study and in round-robin tests involving partners in the European project BELENUS. The test consisted in exposing coated and uncoated samples at 600 °C for 24, 168, 500, 1000, 2000, and 8000 h in an atmosphere consisting of 5% O_2_ + 20% H_2_O + N_2_ (bal) at a gas flow velocity of 0.1 cm/s. Before the test, 2.0 mg/cm^2^ of KCl was deposited on the samples by spraying a saturated KCl(aq) solution and then drying in a stream of air. However, in the 8000 h exposure, 3.0 mg/cm^2^ KCl was deposited. The samples were positioned vertically in the tubular furnace and parallel to the flowing gas. In order to minimize volatilization of KCl from the samples, a crucible containing 3 g of KCl was placed immediately upstream, at the same temperature as the samples. The experiment was designed in order to ascertain that KCl(s) is present on the samples throughout the exposure period (see ref (31) for a detailed description of the test setup). At least two samples per system and under conditions were exposed. The tests were performed isothermally using INTA’s self-designed rig, and the samples were cooled to room temperature before weighing. Further details on the exposure setup can be found in ref (32).

### 2.3 Characterization Techniques

After each exposure, the surface of the coated and uncoated samples was first characterized by X-ray diffraction (XRD) from 20° to 120° in 2θ, with a 0.5° step and a 2 s holding time, using a Panalytical X’Pert, Cu Kα_1_ (1.5406 Å). Cross sections of the exposed samples were metallographically prepared. The cross sections were characterized by field emission scanning electron microscopy (FESEM) using two different microscopes: (1) Thermo Fischer Scientific Apreo C LoVac equipped with an electron dispersive spectroscopy (EDS) of Oxford Instruments Aztec and (2) Carl Zeiss Merlin with a EDS detector for analysis of the energy of scattered X-rays X-Max (20 mm^2^) with a silicon drift detector (SSD) from Oxford Instruments and resolution in energy below 123 at 5.9 eV of Mn Kα.

The crystallographic and grain characteristic data for 168 and 8000 h exposed samples was obtained using electron backscatter diffraction (EBSD) on a TESCAN GAIA3 dual-beam instrument operated at 20 kV with a scanning step size of 0.1 μm. During analysis, the sample was tilted at 70°.

## 3 Results

### 3.1 As-Deposited Coating

**Figure 1:**
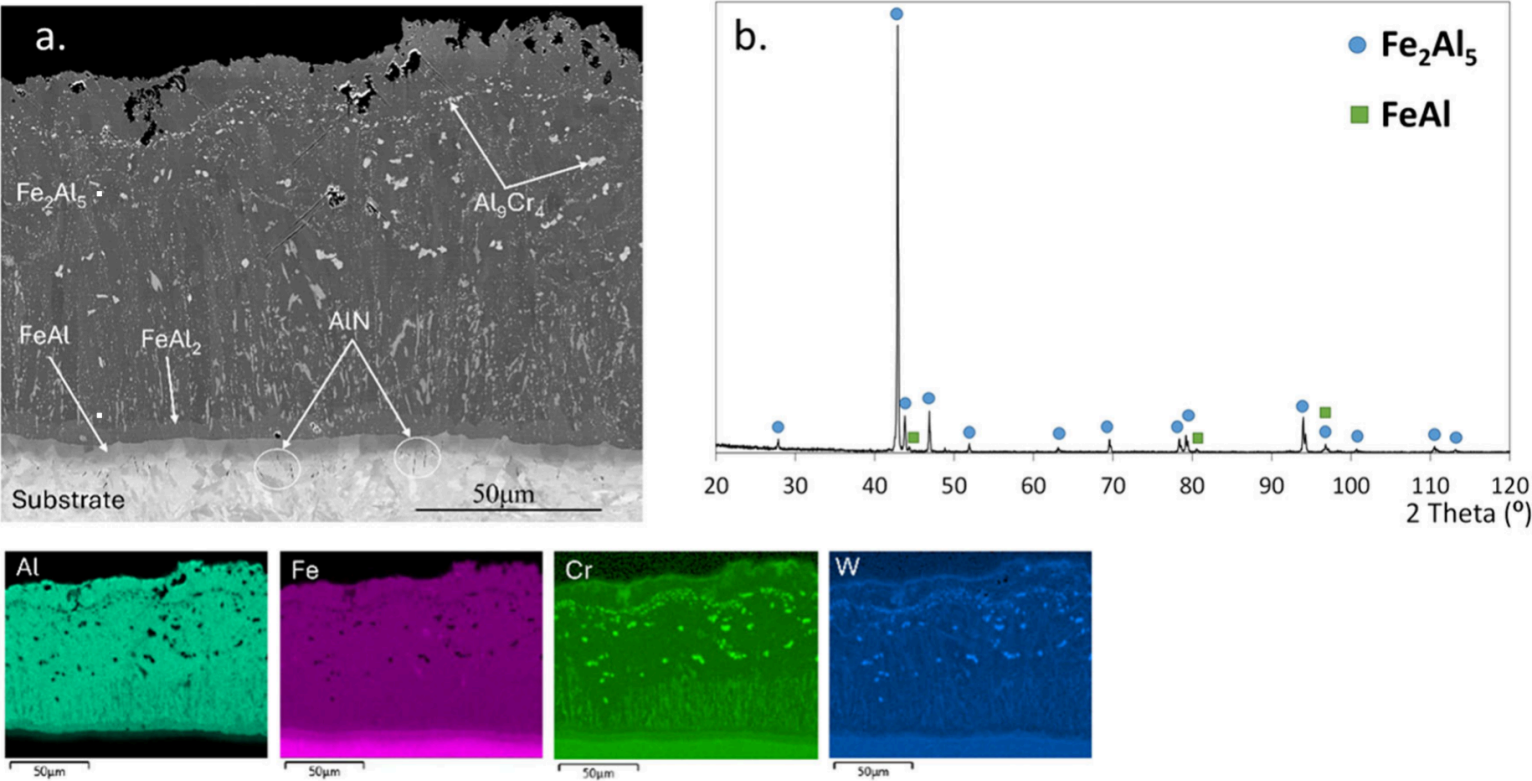
As-deposited slurry aluminide coating on SVM12 before exposure: (a) cross-section FESEM image and EDS element maps; (b) X-ray diffraction pattern.

The slurry deposited aluminide coating has been thoroughly characterized.^14^ It exhibits three main intermetallic zones: a thick outer layer corresponding to Fe_2_Al_5_ with 55 wt %–70 at. % of Al at the surface and two thin inner layers consisting of FeAl_2_ and FeAl (Figure 1). The presence of Fe_2_Al_5_ and FeAl was confirmed by both XRD and electron diffraction. Between the two layers, i.e., FeAl and Fe_2_Al_5_, a thin layer of FeAl_2_ was observed. Although this layer was not confirmed by XRD, it is expected to form under the current conditions; see the FeAl phase diagram.^33^ The coating was heat-treated under Ar, but the residual O_2_ generates a thin Al_2_O_3_ scale (not visible in the FESEM) on the coating. This depletes the subjacent Fe_2_Al_5_ in Al, partially transforming it to FeAl which forms “islands” in the top part of the coating, likely causing the observed low-intensity FeAl XRD peaks observed. The overall coating thickness of about ∼100 μm is rather homogeneous. The bright particles within the Fe_2_Al_5_ layer (Figure 1) consist of Al_9_Cr_4_ precipitates containing dissolved W. Al nitride (AlN) precipitates were detected at the coating/substrate interface; see, e.g., regions marked by white circles. Some thickness-through cracks are present as a result of the thermal expansion mismatch between the different phases. However, it was shown that the cracks do not cause substrate degradation in several harsh atmospheres under which the coating has been tested, such as steam,^34^ coal combustion gases,^20^ and molten salts.^35,36^

### 3.2 Corrosion during Laboratory Exposures in a Model Biomass-Combustion Environment at 600 °C

**Figure 2:**
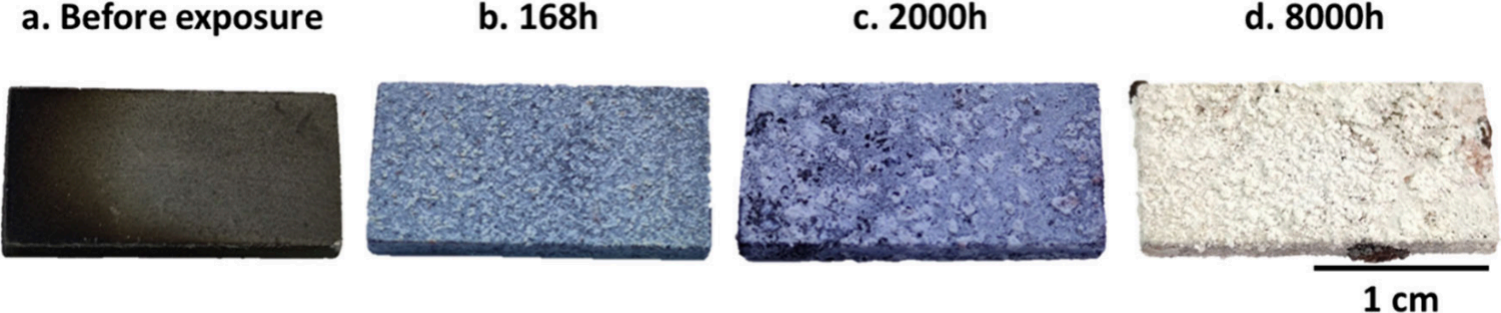
Images of the samples (a) before exposure and (b, c, and d) 168, 2000, and 8000 h of exposure in the laboratory biomass model atmosphere at 600 °C. Remains of KCl salts are visible.

Figure 2 shows the images of the exposed samples after 168, 2000, and 8000 h in the laboratory biomass model atmosphere at 600 °C. The surface could not be directly compared with the surface of the nonexposed samples because of the presence of remaining KCl salt after exposure.

#### Morphology Overview and XRD Analysis

**Figure 3:**
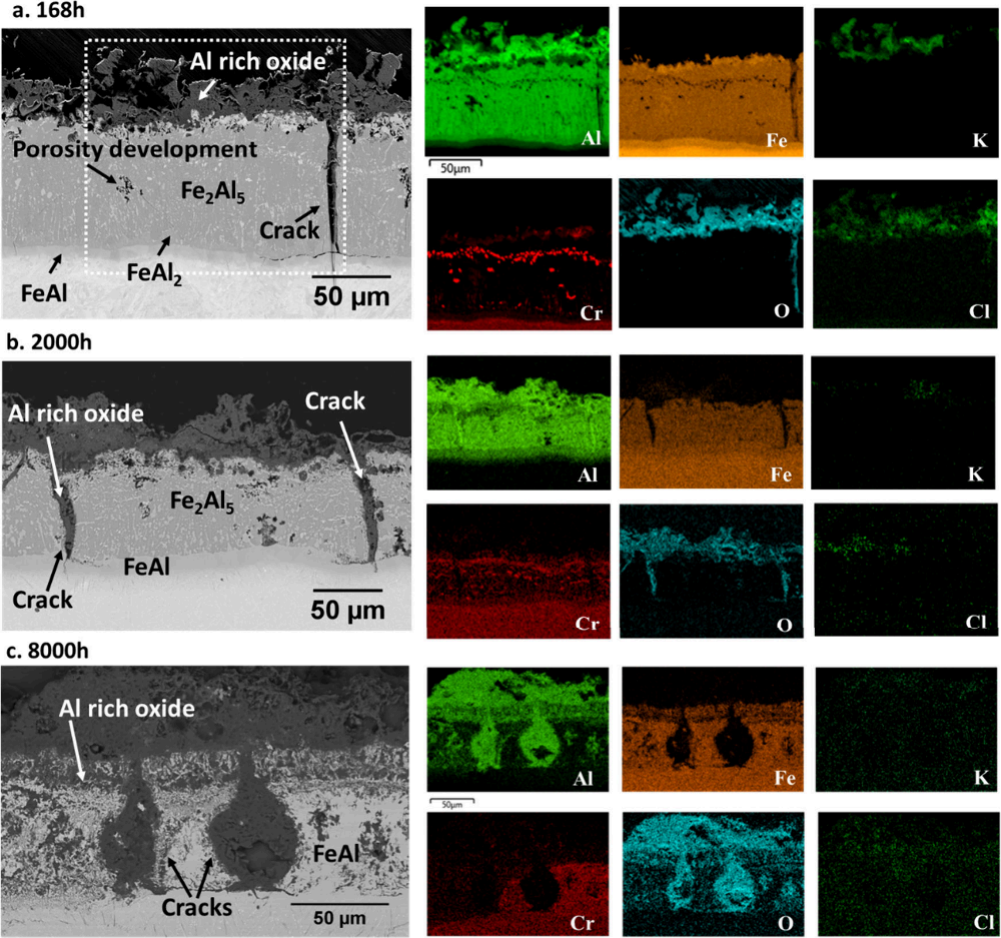
FESEM imaging and EDX maps of the aluminide coating after exposure in the laboratory biomass model atmosphere at 600 °C for 168, 2000, and 8000 h.

**Figure 4:**
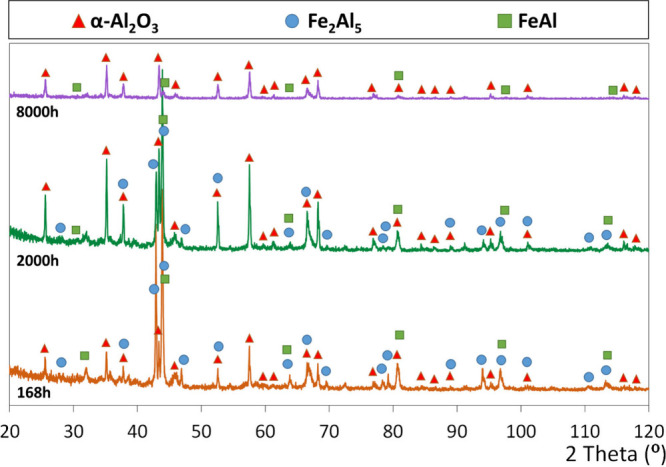
X-ray diffraction patterns of the aluminide coating on SVM12 after (a) 168, (b) 2000, and (c) 8000 h at 600 °C in the biomass model atmosphere.

Surprisingly, a thick, irregular Al-rich oxide scale was observed after 168 h, as seen in the EDS element map in Figure 3a. The XRD pattern (Figure 4a) exhibited relatively high intensity peaks corresponding to α-Al_2_O_3,_ together with peaks attributed to the coating’s intermetallic phases Fe_2_Al_5_ and FeAl. There were no significant unidentified XRD peaks. The Al_2_O_3_-rich layer was porous and had a maximum thickness of 50 μm. It is noted that such thick, fast growing Al_2_O_3_ scales did not develop when the same coating was exposed to steam or to model atmospheres mimicking coal combustion or even biomass/coal co-combustion^37^ for significantly longer periods. Some porosity was present within the aluminum coating, likely caused by very high rates of Al depletion as the Al_2_O_3_ scale develops on the coating surface.

The coating microstructure after 2000 h of exposure was similar to that observed after 168 h (Figure 3). However, the FeAl layer increased slightly from approximately 4 to 6 μm, and the FeAl_2_ layer disappeared. The cracks originally present in the coating widened, possibly as a result of Al_2_O_3_ growth on the crack surface, and the level of porosity in the coating increased significantly (Figure 3b). At this stage, the α-Al_2_O_3_ XRD peaks grew in intensity, both relative to the peaks representing the intermetallics in the coating, and compared to the α-Al_2_O_3_ peaks observed after 168 h, indicating further growth of the α-Al_2_O_3_ scale (Figure 4b). Finally, after 8000 h, transformation/degradation of the coating was evident, again related to Al depletion as the α-Al_2_O_3_ scale continued to grow (Figure 3c). At this stage, the coating mostly consisted of FeAl as evidenced by XRD (Figure 4c), with the peaks representing Fe_2_Al_5_ being absent. Also, the degree of porosity in the coating was very high. In addition, the original FeAl zone adjacent to the substrate grew in thickness indicating that coating–substrate interdiffusion is a cause of further Al loss from the coating. The cracks originally present in the coating widened significantly after 8000 h.

#### High-Resolution FESEM/EDS

Other than Al and O, EDS analysis in Figure 3 indicated the presence of K (from 1 to 12 wt %/0.5 to 8 at. %) and Cl (from 1 to 4 wt %/0.6 to 2.1 at %), depending on position. Potassium is concentrated in the outer part of the Al_2_O_3_ scale while chlorine is concentrated close to the scale/aluminide interface.

**Figure 5:**
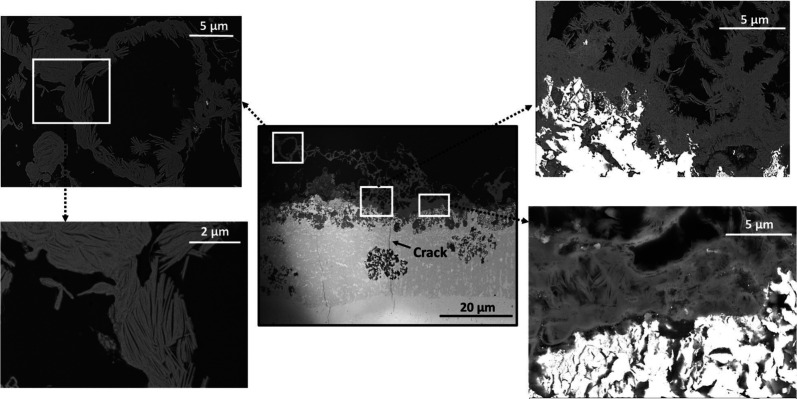
High-magnification FESEM cross-section imaging of the Al_2_O_3_ scale formed on the aluminide coating after 2000 h at 600 °C under a biomass model atmosphere.

Figure 5 shows high-resolution FESEM images of cross sections from different areas in the scale formed after 2000 h. Round features can be observed, likely resulting from the growth of Al_2_O_3_ on the surface of undiffused Al particles, which remained on top of the coating after the heat treatment. The image reveals that the Al_2_O_3_ scale contained aggregated 1–2 μm length needles and whiskers, forming a porous layer. Other regions on the Al_2_O_3_ scale appear to be made up of equiaxed crystals.

**Figure 6:**
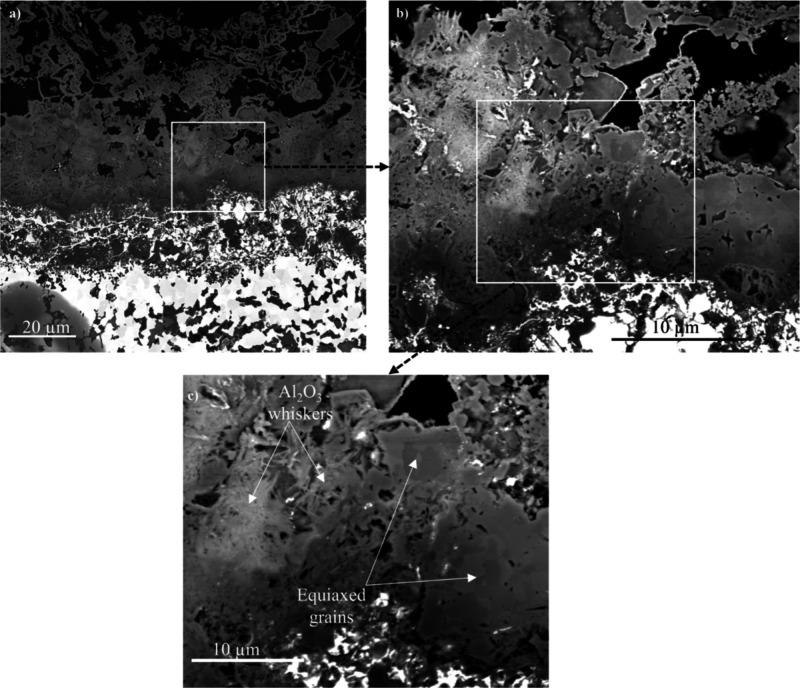
(a) FESEM/BSE cross-sectional image of the oxide scale formed on the aluminide coating after 8000 h at 600 °C under biomass model environment. (b, c) High-magnification images of the region indicated in (a).

Figure 6a shows a corresponding image of the oxide scale after 8000 h. The high-magnification images in Figure 6b,c show the inner part of the Al_2_O_3_ scale and the underlying FeAl. Two distinct oxide scale morphologies were observed, equiaxed grains on one hand and aggregates of much smaller whiskers on the other hand. The equiaxed grains are considered to consist of α-Al_2_O_3_ (see below).

#### SEM/EBSD

**Figure 7:**
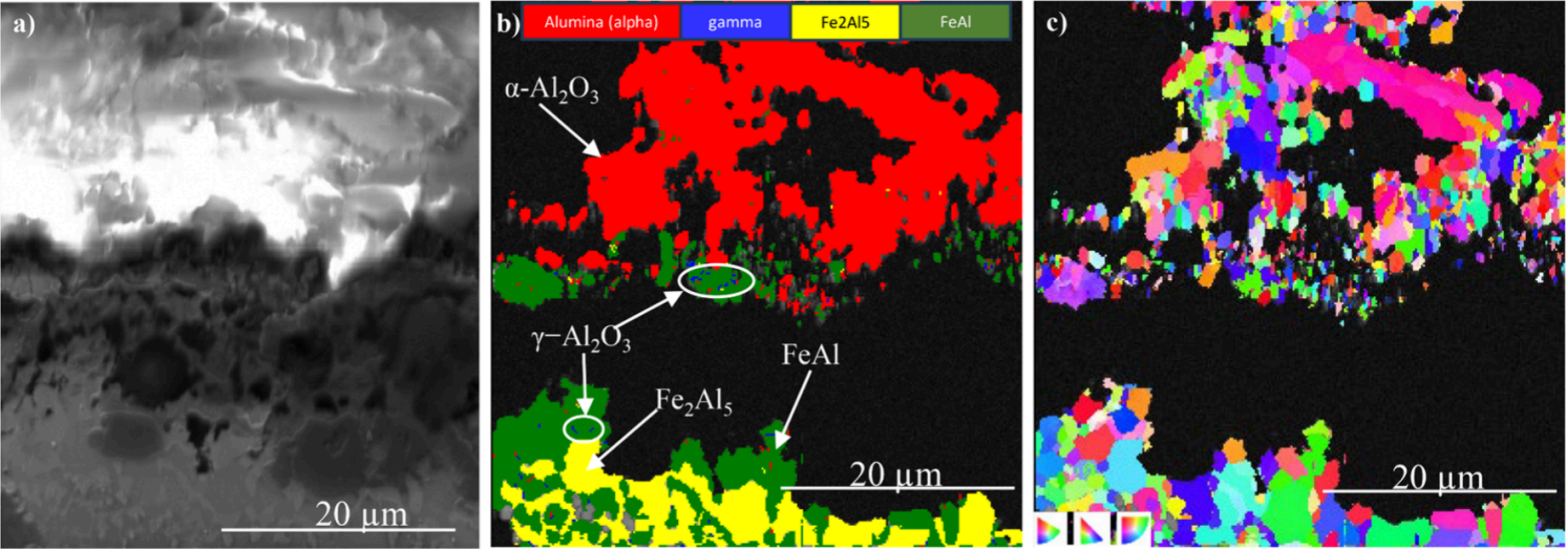
SEM/EBSD cross-section imaging of the oxide scale formed on the aluminide coating after exposure in biomass model atmosphere at 600 °C for 168 h: (a) electron image, (b) phase map, and (c) IPF map.

The cross section in Figure 7 illustrates SEM-imaging (a) and EBSD maps revealing crystalline phases (b) and grain microstructure after 168 h of exposure. The BSE image shows charging of the Al_2_O_3_-rich region, the phase map (Figure 7b) showing that the same region mainly consists of α-Al_2_O_3_. The Al_2_O_3_ scale contains large pores and has delaminated from the coating, possibly during sample preparation. FeAl was identified beneath the Al_2_O_3_ layer together with Fe_2_Al_5_. A small number of γ-Al_2_O_3_ particles were embedded in the FeAl-dominated top part of the coating, beneath the α-Al_2_O_3_ layer (highlighted in Figure 7b). The inverse pole figure (IPF) map in Figure 6c shows the crystallographic orientation on the Al_2_O_3_ scale and in the coating. It reveals that the Al_2_O_3_ scale contains equiaxed grains with an average grain size of about 1.5 μm. The “black” areas in the IPF map correspond to pores and to areas dominated by the Al_2_O_3_ whiskers (see Figures 5 and 6).

**Figure 8:**
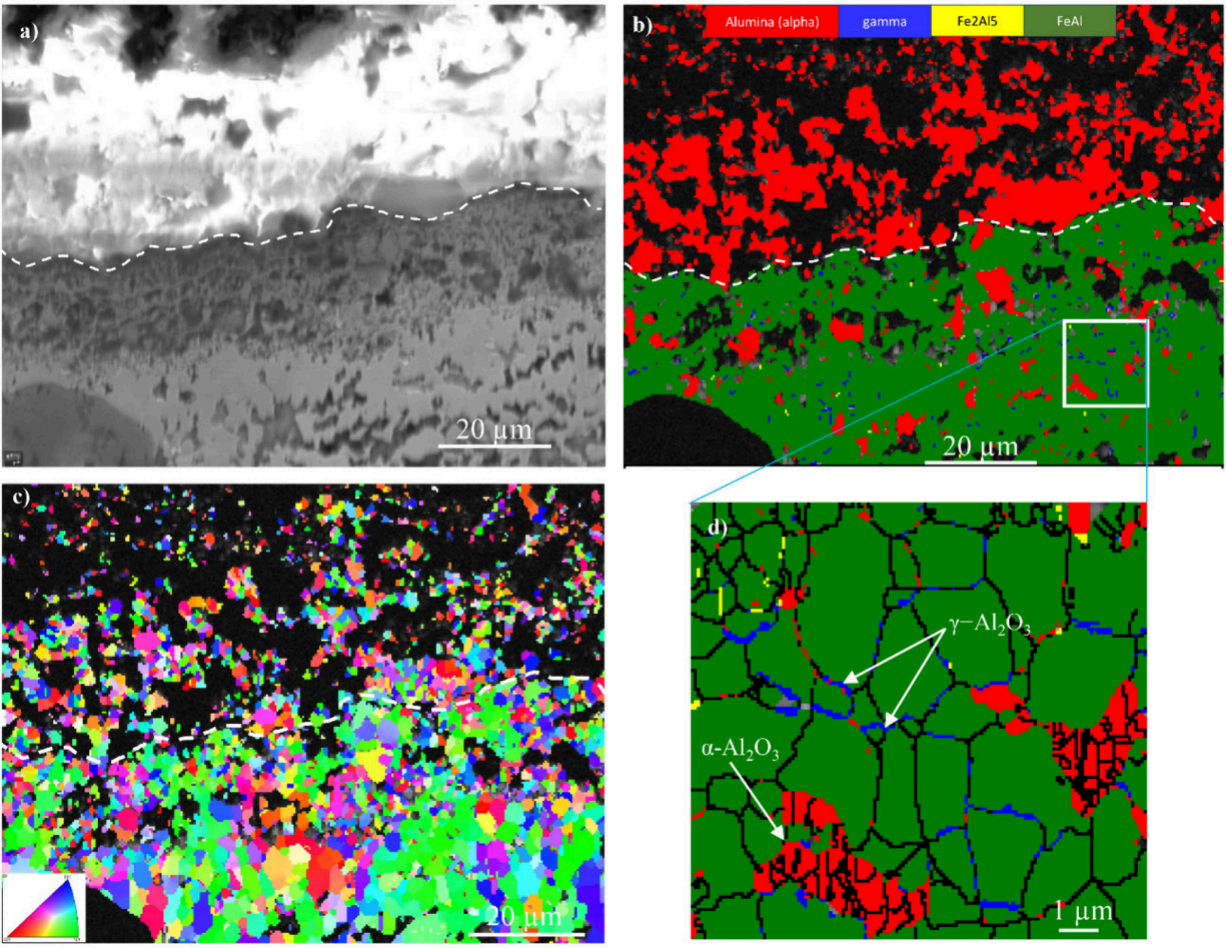
SEM/EBSD results from the area shown in Figure 6a showing (a) SEM-BSE cross-section image, (b) phase map, (c) IPF map, and (d) high-magnification phase map revealing γ-Al_2_O_3_ in the grain boundaries of the coating after exposure in the biomass model atmosphere at 600 °C for 8000 h. The hatched lines in parts a–c indicate the scale/aluminide interface.

After 8000 h exposure, a thick and fractured α-Al_2_O_3_ scale was formed on the coating (Figures 6 and 8). Again, the BSE image shows that the Al_2_O_3_ layer is charging, and the phase map reveals the presence of α-Al_2_O_3_ (Figure 8a,b). In this case, α-Al_2_O_3_ not only was present in the oxide scale but also was detected within the subjacent aluminide (FeAl) layer. The latter α-Al_2_O_3_ grains appear to be located at the pores in the top part of the FeAl layer. Similar to the 168 h results (Figure 7), the α-Al_2_O_3_ grains in the scale were equiaxed with an average grain size of 1.5 μm (Figure 8c), and small γ-Al_2_O_3_ grains were present in the FeAl grain boundaries (see Figures 8b and 8c).

## 4 Discussion

### 4.1 General Corrosion of the Aluminide Coating

FESEM/EDS imaging and X-ray diffraction analysis (Figures 3 and 4) evidence that aluminum in the coating is preferentially oxidized, resulting in formation of a thick α-Al_2_O_3_ scale and converting the top of the Fe_2_Al_5_ coating to FeAl. The sum reaction for the corrosion process is thus
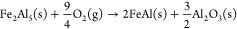
1The role of KCl in this reaction is discussed below. The reaction was rapid during the first 168 h, and the growth of the Al_2_O_3_ layer then continued at a slower pace (cf. Figures 3 and 4). After 8000 h, the remaining aluminide coating was fully converted to FeAl. The fast Al_2_O_3_ growth is in marked contrast to the oxidation behavior of iron aluminide coatings in the absence of KCl, which is reported to result in formation of very thin Al_2_O_3_ layers.^14,34^ Also, the formation of α-Al_2_O_3_ at 600 °C is unexpected. While the Al_2_O_3_ scale is adherent, it contains large pores/cracks, which might undermine its protectiveness. Imaging after 168, 2000, and 8000 h reveal oxide-filled cracks in the coating which reach the steel substrate (cf. Figure 3). However, the lack of evidence for corrosion of the stainless-steel substrate beneath the cracks implies that they self-healed and did not become paths for corrosive species to the steel substrate.

### 4.2 Formation of α-Al_2_O_3_

Industrially, all Al_2_O_3_ polymorphs are prepared by thermal decomposition of Al hydroxides or aluminum oxyhydroxides (AlOOH). The starting materials come in several polymorphic forms, and with increasing temperature, each Al_2_O_3_ precursor generates its characteristic “sequence” of Al_2_O_3_ phases, the transformation process ending with α-Al_2_O_3_. While the industrial production of α-Al_2_O_3_ powders is carried out at 1100–1200 °C, it is noted that one of the Al oxyhydroxide polymorphs, the hexagonal diaspore (α-AlOOH), transforms directly to α-Al_2_O_3_ upon heating already at 450–600 °C.^2^

The temperatures normally required to spontaneously form α-Al_2_O_3_ scales on materials (>900 °C) are in line with the high temperatures used in the industrial production of α-Al_2_O_3_. The somewhat lower temperature (800 °C) reported for formation of protective α-Al_2_O_3_ layers on FeCrAl’s^38^ has been suggested to be due to the presence of the isomorphic Cr_2_O_3_ (eskolaite) in the growing scale. An example of corundum formation at even lower temperature comes from INTA’s laboratory where it was observed that when heating the gibbsite form of Al hydroxide at 650 °C under pure steam, χ-Al_2_O_3_ was detected after 24 h by XRD while α-Al_2_O_3_ was detected after 1250 h.^34^ As mentioned in the Introduction, χ-Al_2_O_3_ was found by electron diffraction on the aluminide coating exposed to steam at 650 °C.^34^

### 4.3 Role of KCl

Alkali chlorides are powerful high-temperature corrosion accelerators toward a variety of materials. With Cr_2_O_3_ forming alloys at 600 °C, the combination of O_2_ and KCl tends to cause breakaway corrosion due to formation of K_2_CrO_4_ which depletes the alloy surface in Cr and because of the formation of CrCl_2_ and/or FeCl_2_ at the scale/alloy interface which causes further scale damage.^39^ In a similar way, the rapid KCl-induced corrosion of steel at 400–500 °C is reported to involve formation of FeCl_2_ at the scale/alloy interface while potassium (formally K_2_O) was associated with hematite at the scale/gas interface.^40^ In both these cases, the redistribution of chlorine and potassium in the scale was attributed to KCl taking an active part in the electrochemical oxidation of the alloy. In the presence of O_2_, Al_2_O_3_ is much less prone to react with alkali than Cr_2_O_3_, implying that Al_2_O_3_ forming materials are promising for high-temperature, alkali-containing environments. However, an Al_2_O_3_ forming FeCrAl alloy exposed to KCl at 600 °C was reported to suffer rapid corrosion, and the resulting corrosion product layer was not enriched in Al.^41^ The poor corrosion behavior was attributed to the slow formation of a protective Al_2_O_3_ layer at this temperature. Moreover, preoxidation of the alloy at higher temperature to form a continuous Al_2_O_3_ film only provided temporary protection. While both the iron aluminide coating and the FeCrAl’s thus suffer rapid corrosion in the presence of KCl and O_2_, the iron aluminide coating exhibits preferential oxidation of aluminum and forms a thick α-Al_2_O_3_ scale, showing that its corrosion behavior is fundamentally different from the FeCrAl’s.

### 4.4 Rapid Al_2_O_3_ Scale Growth

While the formation of an α-Al_2_O_3_ scale at the relatively low temperature of 600 °C is surprising in itself, the rapid growth of that scale is even more unexpected, as alumina and especially α-Al_2_O_3_ scales on alloys are known for their slow growth. Indeed, the slow growth of α-Al_2_O_3_ scales explains the preference for Al_2_O_3_ forming alloys before Cr_2_O_3_ forming alloys in applications at much higher temperatures (>1000 °C). It is argued that corrosion morphology can provide clues for rationalizing this unexpected behavior. Especially, the prominence of Al_2_O_3_ whiskers in the scale is considered important. Protective α-Al_2_O_3_ layers on alloys grow by a solid-state process, and the characteristically slow kinetics are directly related to the very low diffusivity of Al^3+^ and O^2–^ ions in the crystal.^3^ In contrast, the formation of Al_2_O_3_ whiskers shows that rapidly diffusing Al-containing species are present on the corroding iron aluminide surface. Indeed, the rapid diffusion of Al^3+^ likely involves gaseous species or species in the adsorbed state and not ions in the solid state.

As noted above, exposure of steel and stainless steel to KCl–O_2_–H_2_O environments in the same temperature range results in formation of transition metal chlorides (CrCl_2_ or FeCl_2_) at the scale/alloy interface, while potassium accumulates at the scale/gas interface. Because of charging, it was difficult to map the distribution of K and Cl in the Al_2_O_3_ layer. However, the elemental maps for the 2000 h samples (Figure 3b) show that potassium is concentrated at the top of the scale, while chlorine is found close to the aluminide/oxide interface, similar to the reports on stainless steel and steel. This implies that the reaction of KCl with the aluminide coating is analogous to the reactions with stainless steel and steel. Thus, it is suggested that the corrosion reaction involves the formation of AlCl_3_ at the scale/aluminide interface. The formation of FeCl_2_ is considered unlikely because it is far less favored by thermodynamics than AlCl_3_. It is noted that unlike FeCl_2_ and CrCl_2_, AlCl_3_ does not form a condensed phase at the experimental temperature (the critical point of AlCl_3_ is at 353 °C and 26 bar), meaning that it will tend to diffuse toward the scale surface in gaseous form. At the experimental temperature, gaseous AlCl_3_ is partly dimerized, forming a mixture of AlCl_3_(g) and Al_2_Cl_6_(g). In contact with water vapor at 600 °C, AlCl_3_(g) reacts spontaneously according to
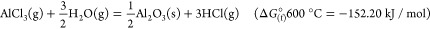
2Databases from the HSC Chemistry 10 were used. Reaction 2 was studied by Park et al.^42^ at 300–700 °C, using a flow reactor at atmospheric pressure. According to ref (42), the reaction first results in formation of gaseous Al hydroxychloride monomers (e.g., AlCl_2_(OH)) which then go on to form larger molecules by condensation. With growing molecular size, the vapor pressure decreases, eventually resulting in formation of solid nanosize particles. The solid product was reported to be amorphous and contained significant concentrations of both chlorine and hydroxide, corresponding to Al_2_O_3(−1/2)(*x*+*y*)_Cl_*x*_(OH)_*y*_.

It is argued that the Al_2_O_3_ whiskers observed in the present study have formed by processes similar to that described by ref (42). In this scenario gaseous AlCl_3_ generated at the scale/alloy interface, as part of the corrosion process, reacts with water vapor within the porous scale to form gaseous Al hydroxychlorides which eventually form the Al_2_O_3_ whiskers. It is noted that the corrosion processes investigated in the present paper are very slow in comparison to the AlCl_3_ hydrolysis described in ref (40). Hence, while the solid product in ref (40) is formed from a highly supersaturated gas, the corresponding reactions on the corroding aluminide surface are expected to involve a lower degree of supersaturation. It is suggested that this explains the formation of Al_2_O_3_ needles in our corrosion experiment rather than nanospheres as in ref (40). Moreover, the solid product in ref (40) was not aged, likely explaining the relatively high concentration of chlorine and hydroxide reported even at 600 and 700 °C. In contrast, the Al_2_O_3_ whiskers observed in the present study were exposed at 600 °C for 168–8000 h, implying that they contain comparatively less chloride and hydroxide. The dominance of Al_2_O_3_ and oxygen in EDX (Figure 3c) and the strong charging during SEM imaging indicate that the whiskers observed consist of Al_2_O_3_. Indeed, the absence of evidence for crystalline aluminas other than α-Al_2_O_3_ in the XRD patterns suggests that the whiskers may consist of α-Al_2_O_3_. However, the question about the phase identity of the whiskers is left open for now. It will be addressed in a forthcoming TEM investigation.

### 4.5 Two Forms of Al_2_O_3_ in the External Scale

Together with the Al_2_O_3_ whiskers, the main scale component is α-Al_2_O_3_ in the form of equiaxed grains with a diameter of about 1.5 μm (see Figures 6b, 7b,c, and 8b,c). The coexistence of equiaxed α-Al_2_O_3_ grains and Al_2_O_3_ whiskers after all exposure times begs the question how the two forms of Al_2_O_3_ are related. Are the Al_2_O_3_ whiskers converted to equiaxed Al_2_O_3_ grains with time? If so, what is the driving force? Or have the two forms of Al_2_O_3_ formed in parallel by different processes? These and other questions must be left unanswered for now and will be addressed by future work.

### 4.6 Internal and External Formation of Al_2_O_3_

The XRD evidence for α-Al_2_O_3_ in the scale after all exposure times is undisputable (Figure 4), and the prevalence of this phase was also undoubtedly demonstrated by the phase maps and IPF maps in Figures 7b,c and 8b,c. In contrast, γ-Al_2_O_3_ was not identified by XRD but was detected within the aluminide coating by SEM/EBSD (Figures 7b and 8d). It is argued that α-Al_2_O_3_ in the oxide scale and γ-Al_2_O_3_ in the interior of the coating were formed by different routes, i.e., that γ-Al_2_O_3_ was formed by internal oxidation of iron aluminide while α-Al_2_O_3_ in the external scale was formed on the coating surface by hydrolysis of gaseous AlCl_3_. Thus, the formation of α-Al_2_O_3_ via the AlCl_3_/steam route was disallowed during internal oxidation because neither water nor Al chloride can be present in the solid aluminide. Instead, O dissolved aluminum atoms reacted to form γ-Al_2_O_3_.

## 5 Conclusions

The following conclusions can be drawn for this present study:

- Rapid α-Al_2_O_3_ scale growth on an iron aluminide coating in the KCl + O_2_ + H_2_O environment at 600 °C is reported for the first time using several advanced characterization techniques.
- The resulting thick Al_2_O_3_ scale was porous and consisted of both equiaxed α-Al_2_O_3_ grains and randomly oriented aggregated Al_2_O_3_ whiskers.
- The unexpected formation of rapidly growing α-Al_2_O_3_ at relatively low temperature is attributed to the hydrolysis of aluminum chloride generated in the corrosion process.
- The fast growth of the whiskers and equiaxed grains of α-Al_2_O_3_ resulted in a significant Al depletion in the coating and prompted the transformation of Fe_2_Al_5_ to FeAl. Both effects contributed to rapid coating degradation.
- Small amounts of γ-Al_2_O_3_ formed within the aluminide coating by the reaction with O dissolved in iron aluminide, i.e., by internal oxidation.
